# Phenotype-specific estimation of metabolic fluxes using gene expression data

**DOI:** 10.1016/j.isci.2023.106201

**Published:** 2023-02-15

**Authors:** Nicolás González-Arrué, Isidora Inostroza, Raúl Conejeros, Marcelo Rivas-Astroza

**Affiliations:** 1 Universidad Tecnológica Metropolitana, Departamento de Biotecnología, Ñuñoa, Santiago 7800003, Chile; 2 Pontificia Universidad Católica de Valparaíso, Escuela de Ingeniería Bioquímica, Valparaíso, 2362803, Chile

**Keywords:** Cellular physiology, Complex system biology, Omics, Transcriptomics

## Abstract

A cell’s genome influences its metabolism via the expression of enzyme-related genes, but transcriptome and fluxome are not perfectly correlated as post-transcriptional mechanisms also regulate reaction’s kinetics. Here, we addressed the question: given a transcriptome, how unobserved mechanisms of reaction kinetics should be systematically accounted for when inferring the fluxome? To infer the most likely and least biased fluxome, we present Pheflux, a constraint-based model maximizing Shannon’s entropy of fluxes per mRNA. Benchmarked against ^13^C fluxes of yeast and bacteria, Pheflux accurately estimates the carbon core metabolism. We applied Pheflux to thousands of normal and tumor cell transcriptomes obtained from The Cancer Genome Atlas. Pheflux showed statistically significantly higher glucose yields on lactate in breast, kidney, and bronchus-lung tumoral cells than their normal counterparts. Results are consistent with the Warburg effect, a hallmark of cancer metabolism, suggesting that Pheflux can be efficiently used to study the metabolism of eukaryotic cells.

## Highlights

• A novel computational model estimates phenotype-specific fluxomes at the genome-scale
• Results accurately estimate the carbon core metabolism in yeast and bacteria
• Using RNA-seq data, the Warburg effect is predicted in various cancer types

## Graphical abstract

**Figure:**
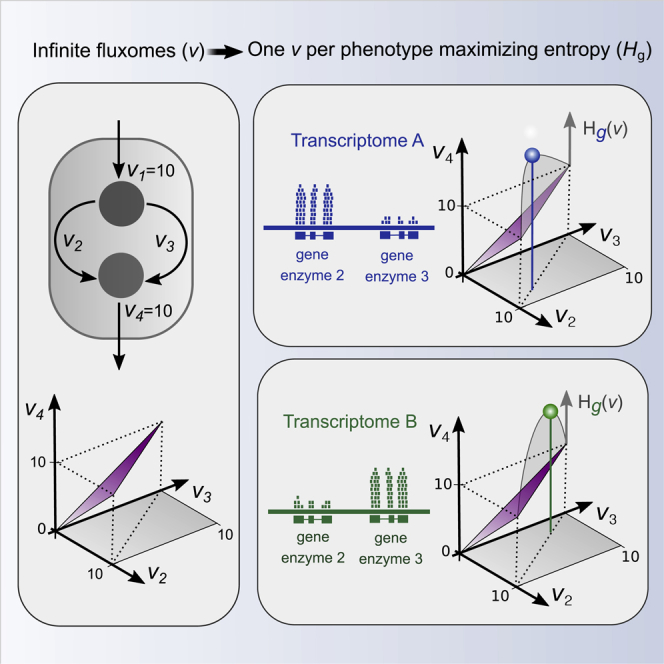

## Introduction

Cells can adapt their metabolism to context-specific conditions by controlling their enzymes production.^1^^,^^2^^,^^3^ This phenomenon has been observed in bacteria and yeasts where tampering with their normal genetic patterns is an effective way to redistribute metabolic fluxes and improve fermentation yields.^4^^,^^5^^,^^6^^,^^7^ Likewise, genetic disorders such as cancer and diabetes are often paired with aberrant distributions of metabolic fluxes.^8^^,^^9^^,^^10^^,^^11^ Anticipating how the genetic expression influences the metabolic state can drive further improvements in the fermentation industry and lead to novel therapies for genetic maladies. These challenges have ushered the development of various mathematical models that infer a cell’s metabolic flux distribution conditioned on its observed gene-expression pattern.^12^^,^^13^^,^^14^^,^^15^^,^^16^^,^^17^^,^^18^^,^^19^ However, translational and post-transcriptional mechanisms, such as enzyme activities and allosteric modulation, also regulate reactions’ kinetics, resulting in the fluxome not being perfectly correlated with the expression levels of their enzyme-related genes.^20^^,^^21^^,^^22^ To cover for these unobserved mechanisms, current mathematical models can only rely on *ad hoc* assumptions,^23^ resulting in inconsistent predictions.^24^^,^^25^ Such ambiguity poses the question: How should the unobserved regulatory interactions between transcriptome and reaction kinetics be systematically accounted for when inferring the fluxome?

Genome-scale fluxomes cannot be computed based on kinetic expressions as these are not fully known for most reactions.^26^^,^^27^^,^^28^^,^^29^ Alternatively, the fluxome space can be constrained by mass and energy conservation principles,^30^^,^^31^ leading to the development of constraint-based models (CBM). In CBMs a steady-state condition is assumed. Consequently, producing and consuming fluxes for each metabolite equate. Applying this assumption to all metabolites in a cell’s metabolic network results in a set of linear constraints defining a solution space of feasible fluxomes.^28^^,^^32^^,^^33^ Measurements of metabolically active molecules, such as RNA, proteins, and metabolites, can be used to inform further reductions of this space.^34^^,^^35^^,^^36^^,^^37^^,^^38^^,^^39^^,^^40^^,^^41^ In this context, transcriptomic patterns are one of the most accessible measurements, as reliable and affordable technologies like micro-arrays and RNA-seq have made gene expression measurements available at genome-wide scales.^42^^,^^43^

Current CBMs using transcriptomic information measure the correlation between fluxome and transcriptome by setting bounds on fluxes bounds, defining a context-specific objective function, or both.^44^ Some methods divide genes between highly and lowly expressed and then select a flux configuration that maximizes the consistency with this classification.^13^^,^^16^^,^^45^ However, these methods required an user-defined threshold to distinguish between both sets of genes. Methods maximizing an *a priori* objective function typically use biomass growth rate, which has proven adequate for unicellular organisms like bacteria and yeast.^46^^,^^47^^,^^48^^,^^49^ However, it may not be appropriate for somatic cells known to maintain stable biomass –e.g., neurons. Alternative objective functions such as maximization of ATP can be used, but these are hard to validate for somatic cells.^50^^,^^51^ Lastly, most methods do not produce a unique solution but only reduce the space of feasible flux configurations from where an arbitrary point is selected to estimate the metabolic state. Other methods can infer a single flux configuration by being formulated as strictly convex optimization problems.^19^^,^^52^ However, many strictly convex functions can maximize the correlation between fluxome and transcriptome but still produce divergent fluxomes.^19^ As a result, currently there is not a method that consistently produces better fluxome estimations that any other alternative.^24^

Here, we used the principle of maximum entropy to develop a mathematical model named Pheflux, which estimates the fluxome conditioned on an organism’s metabolic network and transcriptome. The principle of maximum entropy has been applied to infer genetic interaction networks,^53^ the distribution of growth rates of unicellular organisms,^54^^,^^55^^,^^56^ flux elementary modes,^57^ and fluxomes in the absence of transcriptomic data.^52^^,^^58^ To integrate transcriptomic data, we formulated Pheflux as an optimization problem maximizing Shannon’s entropy^59^ of fluxes per messenger RNA (mRNA). Pheflux formulation stands on two statistical inference arguments stemming from the principle of maximum entropy. First, from an information theory perspective, inferences made in this way correspond to the ones that admit the most ignorance besides prior information.^60^^,^^61^ By being the least biased, the selected fluxome is less susceptible to over-fitting.^62^ Second, from a statistical mechanics perspective, these inferences are the ones that can happen in the greatest number of ways.^63^ Without further information, it is reasonable to assume that all feasible fluxomes can occur. As a result, the selected fluxome is the most likely to be observed. Of interest, we show that Pheflux is equivalent to minimizing the forward Kullback-Leibler divergence between fluxome and transcriptome, providing a framework to argue that this function is the best to measure a statistical distance between both vectors. In addition, we found that Pheflux predictions in bacteria and yeast outperform alternative methods and that it recapitulates the Warburg effect in cancer human cells.

## Results

### Pheflux estimates are less sensitive to thermodynamically infeasible cycles

**Figure 1:**
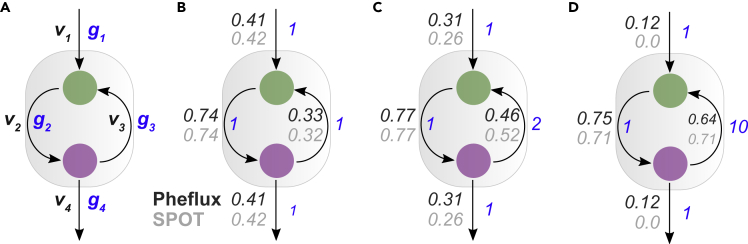
Toy network with a thermodynamically infeasible cycle The network consists of two metabolites and four reactions, where v represents fluxes and g genes’ expression (A) When all reactions have equal gene expression values, Pheflux (dark gray) and SPOT (light gray) produce similar fluxome estimations (B) When $g_{3}$ magnitude doubles every other reaction, SPOT cycles higher flux between metabolites than Pheflux (C) When $g_{3}$ is one order of magnitude higher than any other reaction, SPOT only predicts flux cycling between metabolites, with no flux exchange. Conversely, even in this extreme case, Pheflux predicts flux exchange (D).

To gain intuition about how Pheflux differs from current methods, we compared its predictions to the Simplified Pearson Correlation with Transcriptomic data (SPOT) model.^19^ Like Pheflux, SPOT does not require an objective function with biological meaning and has been highlighted as one of the best methods to predict fluxomes conditioned on transcriptomic data.^19^^,^^64^ We used a toy network model consisting of two metabolites and four reactions. This network includes a thermodynamically infeasible cycle (TIC)^65^ (Figure 1A). We focus on TICs, as although there are several methods to remove them from fluxome estimations^58^^,^^66^^,^^67^^,^^68^^,^^69^ these are still ubiquitous among genome-scale metabolic models^66^ and known to introduce artifacts in the form of spurious high-level flux cycles.^52^ TICs are particularly important when considering that the probability distribution function of genes’ expression follows an exponential decay, i.e., many enzyme-coding genes have low expression levels, and a few are highly expressed.^70^ As a result, if highly expressed genes partake in TICs, this is likely to result in nonsensical fluxome estimations, which is particularly relevant as SPOT, as well as Pheflux, are not warranted to produce TICs free fluxome estimations.

**Figure 2:**
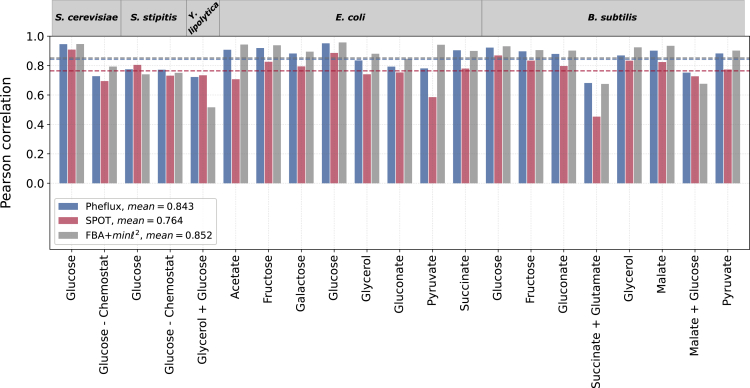
Comparison of Pheflux, SPOT, and FBA+min $\ell^{2}$ estimations to experimental fluxomes of bacteria and yeasts The dataset includes glycolysis and TCA cycle reactions for cultures using single carbon sources and the mixture glycerol-glucose for *Y. lipolytica*, and succinate-glutamate and malate-glucose for *B. subtilis*.

As illustrated by the three study cases in Figures 1B–1D. Pheflux fluxome estimations are less sensitive to TICs than SPOT. In cases 1 (Figure 1B) and 2 (Figure 1C), the genetic expression has little influence in the fluxome inference of both models, probably because of the little difference that exists between the expression level of each enzyme across the network. However, in case 3 (Figure 1D) where reaction 3 has twice the gene expression level of any other reaction, SPOT estimated a biologically infeasible fluxome in which only reactions 2 and 3 carried flux. Pheflux also estimated flux cycling between these two reactions but, unlike SPOT, still predicted exchange fluxes through the intake $\left(v_{1}\right)$ and production $\left(v_{4}\right)$ reactions. These results suggest that Pheflux turns out to be less susceptible to TICs when highly expressed genes are detected. This behavior stems from its foundation, the principle of maximum entropy, which has already been reported to generate more homogeneous fluxomes, avoiding extreme outlier fluxes caused by TICs.^52^

### Central carbon metabolism was predicted with high accuracy

To assess the goodness of fit of Pheflux estimations, we used as benchmark reported flux values of the core carbon metabolism.^71^^,^^72^^,^^73^^,^^74^^,^^75^ We compiled from the literature a dataset encompassing five microorganisms —prokaryotes and eukaryotes— cultured under 21 different conditions (see Table S1). For each condition, this dataset includes a context-specific ${}^{13}C$ derived fluxome of the carbon core metabolism and a transcriptome (generated by either RNA-seq or microarray technologies). We used each transcriptome to condition Pheflux and generated 21 phenotype-specific fluxomes. In addition, we estimated fluxomes using SPOT, and FBA followed by $\ell^{2}$ minimization (FBA+min $\ell^{2}$) as previous reports show that it produces good fluxome predictions despite not considering phenotype-specific data.^19^ We evaluated the goodness of fit of the phenotype-specific fluxomes produced by Pheflux, SPOT, and FBA+min $\ell^{2}$ by comparing them to their corresponding phenotype-specific ${}^{13}C$ derived fluxomes, which is a common validation procedure for CBMs.^24^^,^^55^^,^^64^ We measured the goodness of fit between estimated and experimental fluxes using the Pearson correlation coefficient. Results are presented in Figure 2.

In general, Pheflux yielded an average Pearson correlation value of 0.843 (Figure 2), outperforming SPOT, which resulted in an average value of 0.764. However, Pheflux did not outperformed FBA+min $\ell^{2}\left(\bar{r}=0.852\right)$, which dispenses with transcriptomic information and instead relies on maximizing biomass production. This result is coherent with previous studies reporting that FBA+min $\ell^{2}$ outperforms SPOT and other transcription-based CBMs.^24^^,^^64^ However, using the same transcriptomic information of our *Escherichia coli* study case, Bhadra-Lobo et al. (2020)^64^ reported that SPOT performs better than parsimonious FBA when the uptake rate of the carbon source is missing or speculative. We obtain similar results with Pheflux when compared to FBA+min $\ell^{2}$. Figure S1 shows that Pheflux outperforms FBA+min $\ell^{2}$ when 8 carbon sources (acetate, fructose, galactose, glucose, glycerol, gluconate, pyruvate, and succinate) are left free to be consume from the environment (Figure S1A), and likewise when all possible carbon sources are left open to be consumed from the medium (Figure S1B). A possible explanation for FBA+min $\ell^{2}$ superior performance when the uptake rate is known may stem from the carbon core metabolism being conserved across environmental conditions.^76^ However, the biomass reaction on which FBA+min $\ell^{2}$ relies is fine-tuned for a single source of carbon and may lead FBA+min $\ell^{2}$ astray when various carbon sources are consumed.^50^^,^^77^ In line with this explanation, when more than one carbon source is uptaken, Pheflux outperforms FBA+min $\ell^{2}$. In these cases, Pheflux performance is the best in two out of three cases, and close to the best in the remaining one. The higher variability in the performance of SPOT on these cases may be because of its higher sensitivity to TICs (see Figures 1B and 1C). It has been shown that TICs result in overestimation of fluxes, decreasing the predictive performance of CBMs.^52^ On the other hand, these outcomes suggest that FBA+min $\ell^{2}$ higher performance is particular to the carbon core metabolism. To explore this question, a more informative test would compare estimated and predicted fluxomes at a genome-wide scale. We present such a comparison in the next section.

### Genome-scale fluxome predictions

Current experimental methods do not allow measurements of the fluxome at a genome-scale in a reliable and reproducible manner.^78^ For this reason, we produced genome-wide fluxomes via computational simulations. We used the *optGpSampler* algorithm^79^ to uniformly sample the fluxome space of *E. coli* iJO1366 metabolic network,^71^ generating 11 sets of 1000 samples each. For each sample set, a fraction of the reactions was randomly selected. Gene expression values proportional to their fluxes were assigned to this fraction, and random gene expression values to all others. We varied the selected fraction, λ, between 0 and 1 –in increments of 0.1– to represent uncorrelated and perfectly correlated fluxomes-transcriptomes pairs, respectively. We used the fluxome simulations as a benchmark against which the Pearson correlations of Pheflux and FBA+min $\ell^{2}$ predictions were measured. To test if predictive performance is affected by the selection of the reference reaction set, we computed correlations at genome and carbon core scales.

**Figure 3:**
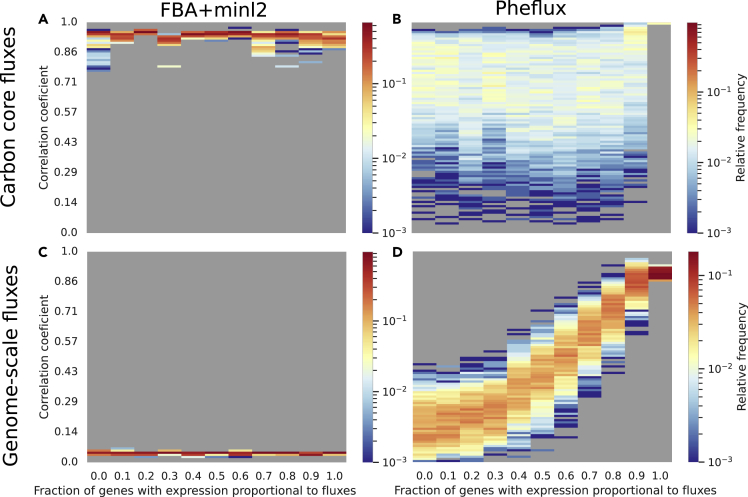
*E. coli* core fluxes predictions under different scenarios. Carbon core fluxes computed by FBA+min l^2^ (A) and Pheflux (B). Genome-scale fluxes computed by FBA+min l^2^ (C) and Pheflux (D).

At the core carbon scale, FBA+min $\ell^{2}$ produced correlations higher than 0.9 in all the scenarios (Figure 3A), outperforming Pheflux in all but the case where 100% of the fluxome correlates with the genetic expression (Figure 3B). FBA+min $\ell^{2}$ high performance is coherent with the results of the previous section as well as with previous publications.^24^^,^^64^

However, FBA+min $\ell^{2}$ advantage in performance did not extend beyond the carbon core test set. At a genome-scale, results show that FBA+min $\ell^{2}$ mean correlation coefficients plump below 0.1 regardless the λ level (Figure 3C). Pheflux performance was always higher than FBA+min $\ell^{2}$ even at λ=0 where gene expression is uninformative of the fluxome state (Figure 3D). It can be inferred that incorporating transcriptomic information via Pheflux improves estimations of the fluxome.

### Pheflux predicted Warburg in cancer cells

To determine whether Pheflux can estimate phenotype-specific fluxome distributions, we evaluated its ability to replicate known metabolic differences between normal and cancer cells at stages I, II, III, and IV.^8^ A hallmark of cancer metabolic reprogramming^9^^,^^80^ is the Warburg effect, also called aerobic glycolysis. The Warburg effect is characterized by an increased glucose uptake rate and subsequent conversion to lactate, regardless of oxygen availability.^81^ We used transcriptomes of Breast (899 tumoral and 95 normal tissue transcriptomes), Kidney (893 tumoral and 128 and normal tissue transcriptomes) and Bronchus–Lung (1036 tumoral and 108 normal tissue transcriptomes) tissues obtained from The Cancer Genome Atlas (TCGA), and we compared their yields of glucose in lactate $\left(v_{lac}/v_{glc}\right)$.

**Figure 4:**
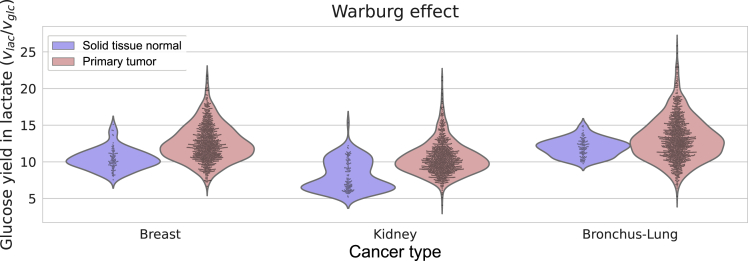
Fluxome estimations for normal and tumor cells for breast, kidney and bronchus-lung tissues. Pheflux estimated higher yields of glucose on lactate $\left(v_{lac}/v_{glc}\right)$ on cancer compared to normal tissues.

**Figure 5:**
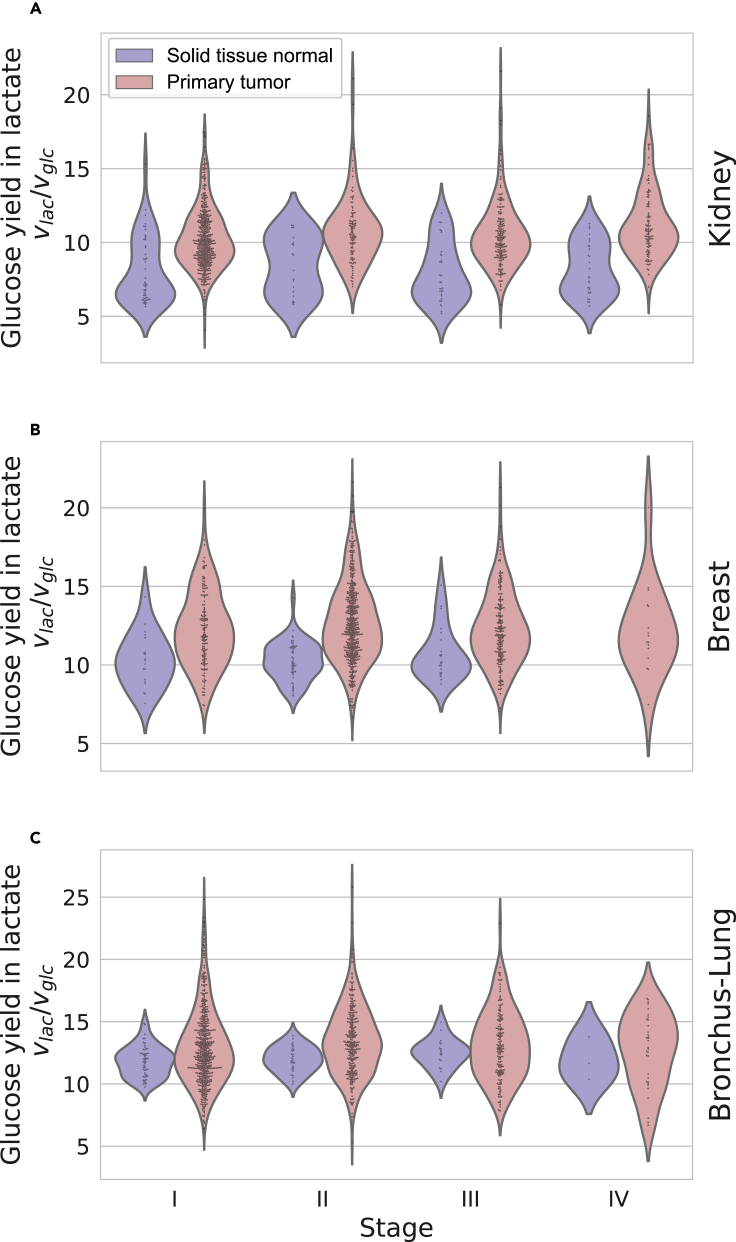
Stage-specific fluxome estimations for normal and tumor cells Results for kidney (A), breast (B) and bronchus-lung (C) tissues. For all cancer types, in all but stage IV (where sample sizes were not big enough to conduct statistical tests), Pheflux estimated higher yields of glucose on lactate $\left(v_{lac}/v_{glc}\right)$ on cancer compared to normal tissues.

As Figure 4 shows, Pheflux fluxome estimations in cancer tissues exhibit, on average, a higher yield of glucose in lactate than in normal tissues ($p-value<1\times 10^{-15}$ for Breast and Kidney cancers, and $p-value<1\times 10^{-4}$ for Bronchus-Lung cancer; Mann-Whitney U test). This distinctive feature can also be observed at cancer developmental stages I, II, and III for all three tissue types (Figure 5; p−value<0.05; Mann-Whitney U test, cancer stage IV was not considered because of lack of sample size), except for stage III of Bronchus-Lung tissues, where there was a significant overlap between the fluxomes of normal and tumoral tissues. These results are coherent with previous reports showing that, on average, tumoral tissues produce higher yields of glucose in lactate,^82^^,^^83^^,^^84^ suggesting that Pheflux can reproduce phenotype-specific fluxomes of cancer cells.

**Figure 6:**
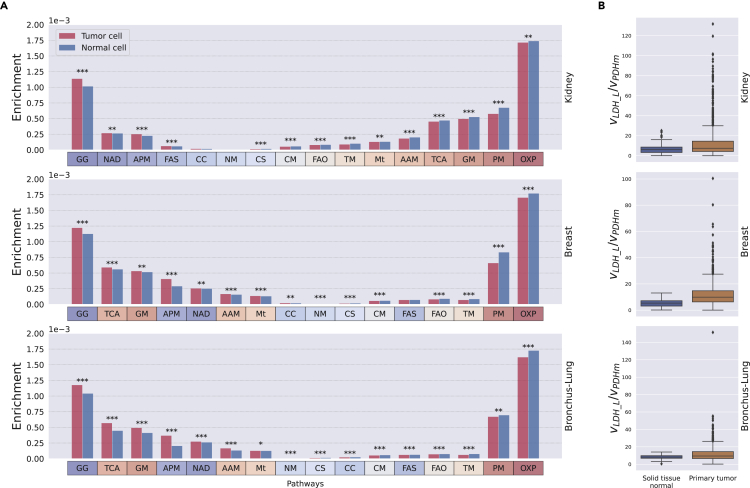
Differential use of metabolic pathways between normal and cancer tissues The relative use of a metabolic pathway (enrichment) between tumoral and normal cells is presented for all three cancer types (A). In all cancer types, the ratio between the fluxes of pyruvate that goes into the Krebs cycle versus lactate –computed as the flux ration between reaction LDH and PDHm– is always greater in tumoral cells (B). The metabolic pathways are coded as: Glycolysis/gluconeogenesis: GG; Oxidative phosphorylation: OXP; Pyruvate metabolism: PM; Glutamate metabolism: GM; Alanine and aspartate metabolism: AAM; CoA catabolism: CC; CoA synthesis: CS; Arginine and proline metabolism: APM; Tryptophan metabolism: TM; Citric acid cycle”: TCA; Nucleotide metabolism: NM; NAD metabolism: NAD; Fatty acid synthesis: FAS; Fatty acid oxidation: FAO; Cholesterol metabolism: CM; and Transport, mitochondrial: Mt. p-values (Mann-Whitney U test) are coded as: <0.0001: ∗∗∗; <0.01: ∗∗; and <0.05: ∗.

The gene expression patterns of various metabolic pathways have been reported to be affected by cancer,^85^ but it is not clear how these differences impact the distribution of metabolic fluxes. We used Pheflux inferences to find differential use of metabolic pathways between normal and tumoral cells. For each pathway, we used as index the average flux magnitude among its reactions (using the reaction pathway-membership reported in Recon3D^86^), and normalized by the sum of all network fluxes magnitudes. This index can be interpreted as the enrichment of such pathway given a distribution of fluxes. Then, we compared the average enrichments of normal and tumoral TCGA samples for each cancer type (Figure 6A). Coherent with previous results,^87^ we found that in all cancer types, oxidative phosphorylation (OXP) is dominant among normal samples, whereas glycolysis/gluconeogenesis (GG) and NAD metabolism are dominant among tumoral samples. However, we found that the tricarboxylic acid cycle (TCA) is dominant among normal kidney samples, but enriched in tumoral breast and bronchus-lung samples. These results do not contradict the Warburg effect as in all cancer types the flux of pyruvate that is diverted toward production of lactate (reaction LDH) is still greater than the flux that diverts pyruvate toward the TCA (reaction PDHm; Figure 6B). We speculate that in bronchus-lung and breast cancers, treatments aimed to downregulate the TCA flux may be of therapeutic value.

### CPU times

**Figure 7:**
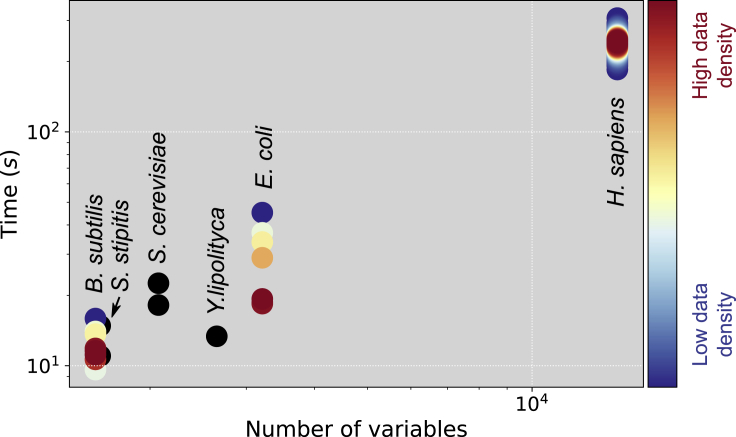
CPU times for different genome-scale metabolic networks Several transcriptomes per species were processed using Pheflux. The data points density for *B. subtilis*, *E. coli*, and *H. sapiens* is color-coded in the blue (low density) to red (high density) range, whereas the non-overlapping data points for *S. stipitis*, *S. cerevisiae* and *Y. lipolytica* are presented in black.

Pheflux CPU times ranged between 30 s and 240 s, depending on the number of variables associated with the size of the metabolic network (Figure 7). For example, metabolic networks with around 3000 variables –*Bacillus subtilis*, *Scheffersomyces stipitis*, *Saccharomyces cerevisiae*, and *E. coli*— the computing times were, on average, 19 s, whereas the large human metabolic network −14000 variables– needed around 240 s to solve. It should be noted that Pheflux was solved without using any specialized algorithms, so that CPU times could be further reduced if a custom implementation is considered.

## Discussion

To infer the fluxome conditioned on a cell’s metabolic network and transcriptome, we developed Pheflux, a novel CBM. Pheflux estimates the fluxome by maximizing Shannon’s entropy of fluxes per enzyme. Such an approach can not completely eliminate the uncertainty of the missing information of reactions’ kinetics, but should, on average, outperform alternative CBMs conditioned on the same prior data because a fluxome inferred according to the principle of maximum entropy corresponds to the one that can happen in the greatest number of ways and with the least amount of unwarranted assumptions. We found support for this hypothesis in the superior performance of Pheflux compared to alternative methods for estimating the fluxomes of various bacteria and yeasts. We further studied Pheflux capacity to infer phenotype-specific fluxomes using thousands of transcriptomes obtained from the TCGA. We found that Pheflux correctly reported higher yields of glucose on lactate on tumor cells compared to their normal counterparts, being this coherent with the Warburg effect, a hallmark of cancer metabolism.

The principle of maximum entropy has been previously used in CBMs to estimate a cell’s fluxome^52^^,^^55^^,^^57^ but, to the best of our knowledge, Pheflux is the first to condition its inferences on transcriptomics. Transcription-based CBMs such as integrative metabolic analysis tool (iMAT),^16^ SPOT, and E-flux2^19^ dispense with the principle of maximum entropy and instead rely on maximizing a mutual relationship between fluxome and transcriptome. Conversely, Pheflux does not require surmising a correlation function as its formulation is equivalent to minimizing a statistical distance –the forward Kullback-Leibler divergence– between fluxome and transcriptome. As such, Pheflux predictions can be interpreted as the fluxome that minimizes the expected excess surprise with regard to the transcriptome. Pheflux does not require or prevent using an *a priori* objective function, which can be useful in cases where such a function is appropriate and easily measurable, for instance, the biomass growth rate for bacteria and yeast. In these cases, measurements of such function can be used as extra constraints of the fluxome space to further improve Pheflux predictions.

The quality of Pheflux inferences is affected by the grade of the data upon which it is conditioned and by the validity of its underlying assumptions. As a CBM, Pheflux requires a fluxome space that faithfully matches the metabolic capacities of the organism under study. For this, it is critical that the genome-scale metabolic network encompasses the reactions, metabolites, and cellular compartments that actually pertain to the organism under study. Once a *bona fide* fluxome space is established, experimentally observed fluxes –e.g., exchange rates of substrates and products– can be used as extra constraints to reduce the fluxome space. Pheflux’s underlying assumptions may also limit the accuracy of its inferences, as the inclusion of transcriptomic information does not yield extra constraints to reduce further the solution space but weights the selection of the most probable fluxome. Pheflux assumes that gene expression is a good proxy of enzyme abundance, but it has been shown that the abundance of enzymes and mRNAs are not perfectly correlated.^21^^,^^22^^,^^88^ In this case, Pheflux estimations may be improved if provided directly with protein concentrations. In addition, Pheflux assumes that all copies of an enzyme carry the same flux. We expect this to be a good approximation for enzymes that move freely within the cell but not for enzymes that are organelle-specific, membrane-attached or otherwise unevenly distributed within the cell. In these cases, more detailed information about enzyme distribution must be provided experimentally. Another limitation of Pheflux is that its predictions are not free of TICs. However, Pheflux can be easily upgraded to prevent this. Fleming et al. (2000)^58^ have shown that the maximization of a functional equivalent to the objective function of Pheflux archives thermodynamically consistent fluxome estimations if all reactions are divided into forward and reverse fluxes. This will increase CPU times by adding extra variables but it is guaranteed to produce TICs free estimations.

CPU times showed that Pheflux could be efficiently applied to large eukaryotic metabolic networks in a computationally efficient manner, even when Pheflux objective function is convex but non-linear. As a result, we expect Pheflux to have a wide range of applications in studies that rely on large metabolic networks, such as RECON3D. These include the study of diabetes,^89^ animal cell cultures,^90^ and metabolic changes in embryo development.^91^ Pheflux will be especially useful to model cell types from multi-cellular organisms, where a biological function may prove hard to come by.

### Limitations of study

In the absence of *in vivo* genome-wide protein concentration data, we used gene expression data as an approximation of enzyme concentrations. However, post-transcriptional regulatory processes may result in mRNA levels not always proportional to their corresponding protein concentrations. As a result, this approximation may limit the accuracy of Pheflux estimations.

## STAR★Methods

### Key resources table

**Table:** 

REAGENT or RESOURCE	SOURCE	IDENTIFIER
**Deposited data**		

*S. cerevisiae* GEM	Mo et al.^72^	Mo et al.,72 iMM904
*S. cerevisiae* RNA-seq transcriptomics	Nookaew et al.^99^	Chemostat and batch, using glucose as car-bon source.
*S. cerevisiae* 13C fluxomics	Papini et al.^73^	Chemostat and batch, using glucose as car-bon source
*S. stipitis* GEM	Liu et al.^105^	iTL885
*S. stipitis* RNA-seq transcriptomics	Papini et al.^73^	Chemostat and batch, using glucose as car-bon source
*S. stipitis* 13C fluxomics	Papini et al.^73^	Chemostat and batch, using glucose as car-bon source
*Y. lipolytica* GEM	Kerkhoven et al.^74^	iYali
*Y. lipolytica* RNA-seq tran-scriptomics	Sabra et al.^100^	Glycerol and glucose as carbon source
*Y. lipolytica* 13C fluxomics	Sabra et al.^100^	Glycerol and glucose as carbon source
*E. coli* GEM	Orth et al.^71^	iJO1366
*E. coli* microarray transcrip-tomic	Gerosa et al.^101^	Eight different carbon sources.
*E. coli* 13C fluxomics	Gerosa et al.^101^	Eight different carbon sources
*B. subtilis* GEM	Oh et al.^75^	iYO844
*B. subtilis* microarray tran-scriptomics	Nicolas et al.^103^	Eight different carbon sources.
*B. subtilis* 13C fluxomics	Chubukov et al.^102^	Eight different carbon sources.
*H. sapiens* GEM	Brunk et al.^86^	Recon3D
Kidney primary tumor and solid tissue normal FPKMs	https://portal.gdc.cancer.gov/	GDC API fields: cases.primary_site: kidney, files.analysis.workflow_type: HTSeq - FPKM
Breast primary tumor and solid tissue normal FPKMs	https://portal.gdc.cancer.gov/	GDC API fields: cases.primary_site: breast, files.analysis.workflow_type: HTSeq - FPKM
Bronchus-Lung primary tu-mor and solid tissue normal FPKMs	https://portal.gdc.cancer.gov/	GDC API fields: cases.primary_site: bronchus and lung, files.analysis.workflow_type: HTSeq - FPKM

**Software and algorithms**		

Pheflux (v 1.0.1)	This paper	https://doi.org/10.5281/zenodo.7383247
Python (v 3.8)	Van Rossum et al.^106^	https://www.python.org/

### Resource availability

#### Lead contact

Further information and requests for resources and reagents should be directed to and will be fulfilled by the lead contact, Marcelo Rivas-Astroza (marcelo.rivas@utem.cl).

#### Materials availability

This study did not generate new unique data or reagents.

### Method details

#### Fluxome space

Given a metabolic network of N reactions and M metabolites, the rate of change of the metabolites’ concentrations over time, $\dot{c}\in R^{M}$, is given by:

(Equation 1) $$Sv=\dot{c}$$

where $v\in R_{+}^{N}$ is the vector of reactions’ fluxes –the fluxome– and $S\in R^{M}\times R^{N}$ the stoichiometric matrix. All fluxes are positive, with reversible reactions been split into forward $\left(v_{i}^{f}\right)$ and reverse $\left(v_{i}^{r}\right)$ reactions, such that the net flux is $v_{i}=v_{i}^{f}-v_{i}^{r}$. Assuming a steady-state condition, Equation 1 reduces to Sv=0. Thermodynamic potentials defining reactions directions or experimentally measured reaction fluxes are incorporated in the form of lower $\left(LB\in R_{+}^{N}\right)$ and upper bounds $\left(UB\in R_{+}^{N}\right)$. All these constraints define the polytope of the fluxome space:

(Equation 2) $$P=\left\{v\in R_{+}^{N}|Sv=0,LB\leq v\leq UB\right\}$$

#### Selection of the most likely and least biased fluxome

For any reaction i in a metabolic network, its flux, $v_{i}$ mmol/gDW/g, is proportional the number of enzyme copies, $q_{i}$. This proportionality can be converted into equality by considering the maximum turnover rate of each enzyme, $k_{i}$ mmol/gDW/h, and a term in the range [0,1], $\eta_{i}$, to account for condition-specific factors affecting the flux kinetics (for instance, inhibition by product) as follows^92^:

(Equation 3) $$v_{i}=\left(k_{i}\eta_{i}\right)q_{i}$$

At present it is not feasible to measure most $\left(k_{i}\eta_{i}\right)$ values under *in vivo* conditions,^92^ but instead they must be statistically inferred. Here, we propose to use the principle of maximum entropy to estimate such values. From Equation 3, $k_{i}\eta_{i}$ can be expressed as:

(Equation 4) $$\left(\eta_{i}k_{i}\right)=\frac{v_{i}}{q_{i}}$$

Equation 4 implies that $\left(k_{i}\eta_{i}\right)$ can be interpreted as the flux per each copy of the enzyme catalyzing reaction i. Assuming that gene expression, $g_{i}$, is a good proxy for $q_{i}$, we replace $q_{i}$ for $g_{i}$ in Equation 4, and then define the probability distribution that is function of v and condition on g, $P_{g}\left(v\right)$, as the relative frequency of each $v_{i}/g_{i}$:

(Equation 5) $$P_{g}\left(v_{i}\right)=\frac{v_{i}/g_{i}}{V}$$

where $V=\sum_{i=1}^{N}\sum_{j=1}^{g_{i}}v_{i}/g_{i}$, and j index each enzyme copy catalyzing reaction i. Assuming that each enzyme copy carries the same flux, the terms $v_{i}/g_{i}$ can be factor out of the inner sum, rendering $V=\sum_{i=1}^{N}g_{i}v_{i}/g_{i}=\sum_{i=1}^{N}v_{i}$. From a statistical mechanics point of view, the $P_{g}\left(v\right)$ that maximizes the Boltzmann’s entropy results in the distribution of $v_{i}/g_{i}$ that can happen in the greatest number of ways.^63^ From an information theory point of view, the $P_{g}\left(v\right)$ that maximized Shannon entropy results in the distribution of $v_{i}/g_{i}$ that requires the least amount of prior information.^59^ Boltzmann and Shannon’s entropies have the same functional form, H, which, when applied to all enzyme copies of each reaction, results in:

(Equation 6) $$H_{g}\left(v\right)=-\sum_{i=1}^{N}\sum_{j=1}^{g_{i}}P_{g}\left(v_{i}\right)\log\;P_{g}\left(v_{i}\right)$$

(Equation 7) $$=-\sum_{i=1}^{N}g_{i}P_{g}\left(v_{i}\right)\log\;P_{g}\left(v_{i}\right)$$

(Equation 8) $$=-\sum_{i=1}^{N}g_{i}\frac{v_{i}/g_{i}}{V}\log\frac{v_{i}/g_{i}}{V}$$

(Equation 9) $$=-\sum_{i=1}^{N}\frac{v_{i}}{V}\log\frac{v_{i}/g_{i}}{V}$$

In this formulation, $H_{g}\left(v\right)$ is a function of the fluxome, v, conditioned on the experimentally observed transcriptome, g. Thus, we defined Pheflux as the selection of v according to the following optimization problem:

(Equation 10) $$\begin{array}{l} \max_{v}H_{g}\left(v\right)\\ \text{subject}\;\text{to}\text{:} \end{array}$$

(Equation 11) v∈P

#### Equivalence with the minimization of the forward kullback-libeler divergence

Equation 10 is equivalent to minimize the forward Kullback-Leibler^93^ between v and g. Factoring the V terms of Equation 9 results in:

(Equation 12) $$H_{g}\left(v\right)=-\frac{1}{V}\sum_{i=1}^{N}v_{i}\log\frac{v_{i}}{g_{i}}+\log\;V$$

A V is a constant, the following equality holds:

(Equation 13) $$\max\left\{H_{g}\left(v\right)\right\}=\max\left\{-\frac{1}{V}\sum_{i=1}^{N}v_{i}\log\frac{v_{i}}{g_{i}}+\log\;V\right\}=\min\left\{\sum_{i=1}^{N}v_{i}\log\frac{v_{i}}{g_{i}}\right\}$$

The rightmost term of Equation 13 is equivalent to minimize the forward Kullback-Leibler divergence^93^ between the probability distribution of the fluxome $P\left(v_{i}\right)=v_{i}/V$ and the probability distribution of gene expression per reaction $Q\left(g_{i}\right)=g_{i}/G$, where G is equal to $\sum_{i}g_{i}$. Thus, the solution of Equation 10 is also the solution of

(Equation 14) $$\begin{array}{cc} \begin{array}{c} \min:\\ v \end{array} & D_{KL}\left(P\|Q\right)=\sum_{i=1}^{N}P\left(v_{i}\right)\log\frac{P\left(v_{i}\right)}{Q\left(g_{i}\right)}\\ \begin{array}{c} s.a: \end{array} & Sv=0\\ & LB\leq v\leq UB \end{array}$$

#### Bioinformatic analyses

The RNA-seq libraries from *S. cerevisiae*, *S. stipitis*, and *Y. lipolytica* (see Table S1) were mapped using STAR 2.5.0a^94^ with default settings. We used as reference genomes the assemblies *S. cerevisiae* sacCer3, *S. stipitis* CBS 6054, and *Y. lipolytica* CLIB122. Gene expression per gene was computed as fragments per thousands of exonic bases per millions of reading mapped. The micro-arrays from *E. coli* and *B. subtilis* were quantile normalized using the limma R package.^95^

#### Computational implementation

As we considered all fluxes non-negative, their lower and upper bounds were assigned 0 and 1000, respectively. Pheflux, SPOT, FBA+min $\ell^{2}$, and Flux sampling were implemented using COBRApy 0.22.1^96^ library in Python 3.8. Pheflux non-linear optimization was done using IPOPT 3.12.3^97^ optimizer through the CasADi 3.5.5^98^ interface. SPOT and FBA+min $\ell^{2}$ optimization were performed using CPLEX 20.1 optimizer and Flux sampling using the *optGpSampler*^79^ implemented in COBRApy.

To perform Pheflux optimization via IPOPT we used the same considerations as Rivas-Astroza & Conejeros (2020),^52^ i.e. we added a small number, ϵ, to each flux to avoid an undefined value of the term $\log\left(v_{i}/\left(g_{i}V\right)\right)$ (Equation 9) when $v_{i}$ or $g_{i}$ are zero. For all computations we used $\epsilon =10^{-8}$. Also, in order to speed up CPU times, we constrained the fluxome space to the subset where the sum of all fluxes (V) are equal to a positive constant. For all Pheflux computations we set V=1000, large enough, to avoid forcing any reaction to reach its bounds.

For SPOT and Pheflux, we assigned gene expression values to each reaction according to their gene-protein-reaction associations.^19^ We used the median value of this set as the gene expression value of any reaction without a reported gene-protein-reaction association.

#### Data sources

We use previously published information^73^^,^^99^^,^^100^^,^^101^^,^^102^^,^^103^ of 5 microorganisms –yeasts and bacteria– grown under 21 culture conditions (see Table S1), which have transcriptomic and fluxomic information that correspond to RNA-seq/microarrays libraries and experimental fluxes using ${}^{13}C$ labeled, respectively. For each microorganism we use the genome-scale metabolic models detailed in Table S1. For all bacteria and yeasts, we consider uptake rates for the genome-scale metabolic network according to the culture media conditions reported by the original authors.

For kidney, breast, and bronchus-lung normal and cancer human tissues, we use Recon3D metabolic network^86^ and transcriptomic data from TCGA (https://www.cancer.gov/tcga). We considered the set of uptake reactions as reported by Shen et al. (2019)^104^ for human tissues.

### Quantification and statistical analysis

In this study, differences between the statistics of tumoral and normal cancer cells were assess using the Mann-Whitney U test. Statistical details can be found in the figure legends, results and method details. All statistical analyses were performed using Python 3.8.

### Additional resources

Pheflux computational implementation and the code used to generate the results used in all figures can be accessed from the following GitHub repository: https://github.com/mrivas/pheflux.

## Data Availability

• This paper analyzes existing, publicly available data. These source references for the datasets are listed in the key resources table. • All original code has been deposited at Zenodo and is publicly available as of the date of publication. DOIs are listed in the key resources table. • Any additional information required to reanalyze the data reported in this paper is available from the lead contact upon request.
