# Supplementary material for: Phenotype-specific estimation of metabolic fluxes using gene expression data
Source: iScience. 2023 Feb 15;26(3):106201. doi: 10.1016/j.isci.2023.106201 (PMC10006673; doi:10.1016/j.isci.2023.106201)
Supplement: Document S1. Figure S1 and Table S1 [file mmc1.pdf]

iScience, Volume *26*

Supplemental information

Phenotype-specific estimation of metabolic fluxes using gene expression data

Nicolás González-Arrué, Isidora Inostroza, Raúl Conejeros, and Marcelo Rivas-Astroza

Supplementary materials

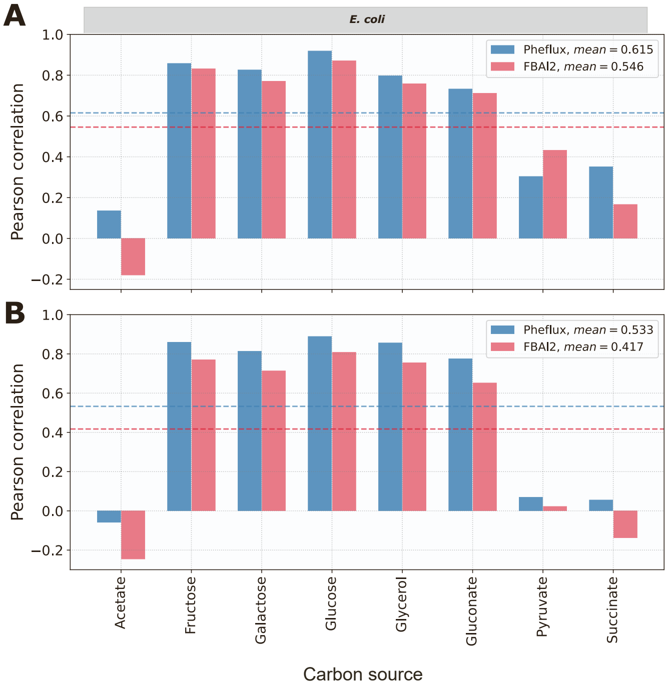

Figure S1: Pheflux and FBA+min ℓ^2 performance in *E. coli* when uptake information is missing. Performance comparison when 8 carbon sources are set as consumption fluxes (A) and when all possible carbon sources are set as consumption fluxes (B). Related to Figure 2.

Table S1: Transcriptome and fluxome data sources considered for all calculations carried on this work. Related to STAR Methods.

Organism & genome-scale metabolic model	Culture conditions	Transcriptomic Data	Fluxomic Data
S. cerevisiae iMM904 [1]	Two conditions —chemostat and batch— supplemented by glucose as carbon source.	Nookaew et al. (2012)[2]: Data measured using RNA-seq technology. Three replicates per condition. Normalized by FPKM.	Papini et al. (2012)[3]: Fluxes measured using ^{13}C labeled. No replicates.
S. stipitis iTL885 [4]	Two conditions —chemostat and batch— supplemented by glucose as carbon source.	Papini et al. (2012)[3]: Data measured using RNA-seq technology. Three replicates per condition. Normalized by FPKM.	Papini et al. (2012)[3]: Fluxes measured using ^{13}C labeled. No replicates.
Y. lipolytica iYali [5]	One condition —mixed culture— supplemented by glycerol and glucose as carbon source.	Sabra et al. (2017)[6]: Data measured using RNA-seq technology. Two replicates. Normalized by FPKM.	Sabra et al. (2017)[6]: Fluxes measured using ^{13}C labeled. No replicates.
E. coli iJO1366[7]	Eight conditions supplemented by glucose, gluconate, galactose, succinate, pyruvate, glycerol, succinate, acetate and fructose, respectively.	Gerosa et al. (2015)[8]: Data measured using microarray technology. Three replicates per condition. Normalized by quantile normalization.	Gerosa et al. (2015)[8]: Fluxes measured using ^{13}C labeled. No replicates.
B. subtilis iYO844 [9]	Eight conditions supplemented by glucose, fructose, gluconate, succinate + glutamate, glycerol, malate, malate + glucose and pyruvate, respectively.	Nicolas et al. (2012) [10]: Data measured using microarray technology. Three replicates per condition. Normalized by quantile normalization.	Chubukov et al. (2013) [11]: Fluxes measured using ^{13}C labeled. No replicates.

References

1. Mo, M. L., Palsson, B. Ø., and Herrgård, M. J. (2009). Connecting extracellular metabolomic measurements to intracellular flux states in yeast. *BMC systems biology* 3.1, pp. 1–17.
2. Nookaew, I., Papini, M., Pornputtapong, N., Scalcinati, G., Fagerberg, L., Uhlén, M., and Nielsen, J. (2012). A comprehensive comparison of RNA-Seq-based transcriptome analysis from reads to differential gene expression and cross-comparison with microarrays: a case study in *Saccharomyces cerevisiae*. *Nucleic acids research* 40.20, pp. 10084–10097.
3. Papini, M., Nookaew, I., Uhlén, M., and Nielsen, J. (2012). *Scheffersomyces stipitis*: a comparative systems biology study with the Crabtree positive yeast *Saccharomyces cerevisiae*. *Microbial Cell Factories* 11.1, pp. 1–16.
4. Liu, T., Zou, W., Liu, L., and Chen, J. (2012). A constraint-based model of *Scheffersomyces stipitis* for improved ethanol production. *Biotechnology for biofuels* 5.1, pp. 1–11.
5. Kerkhoven, E. J., Pomraning, K. R., Baker, S. E., and Nielsen, J. (2016). Regulation of amino-acid metabolism controls flux to lipid accumulation in *Yarrowia lipolytica*. *NPJ systems biology and applications* 2.1, pp. 1–7.
6. Sabra, W., Bommareddy, R. R., Maheshwari, G., Papanikolaou, S., and Zeng, A.-P. (2017). Substrates and oxygen dependent citric acid production by *Yarrowia lipolytica*: insights through transcriptome and fluxome analyses. *Microbial cell factories* 16.1, pp. 1–14.
7. Orth, J. D., Conrad, T. M., Na, J., Lerman, J. A., Nam, H., Feist, A. M., and Palsson, B. Ø. (2011). A comprehensive genome-scale reconstruction of *Escherichia coli* metabolism—2011. *Molecular systems biology* 7.1, p. 535.
8. Gerosa, L., Rijsewijk, B. R. H. van, Christodoulou, D., Kochanowski, K., Schmidt, T. S., Noor, E., and Sauer, U. (2015). Pseudo-transition analysis identifies the key regulators of dynamic metabolic adaptations from steady-state data. *Cell systems* 1.4, pp. 270–282.
9. Oh, Y.-K., Palsson, B. O., Park, S. M., Schilling, C. H., and Mahadevan, R. (2007). Genome-scale reconstruction of metabolic network in *Bacillus subtilis* based on high-throughput phenotyping and gene essentiality data. *Journal of Biological Chemistry* 282.39, pp. 28791–28799.
10. Nicolas, P., Mäder, U., Dervyn, E., Rochat, T., Leduc, A., Pigeonneau, N., Bidnenko, E., Marchadier, E., Hoebeke, M., Aymerich, S., et al. (2012). Condition-dependent transcriptome reveals high-level regulatory architecture in *Bacillus subtilis*. *Science* 335.6072, pp. 1103–1106.
11. Chubukov, V., Uhr, M., Le Chat, L., Kleijn, R. J., Jules, M., Link, H., Aymerich, S., Stelling, J., and Sauer, U. (2013). Transcriptional regulation is insufficient to explain substrate-induced flux changes in *Bacillus subtilis*. *Molecular systems biology* 9.1, p. 709.
